# Spatial normalization of lesioned brains: Performance evaluation and impact on fMRI analyses

**DOI:** 10.1016/j.neuroimage.2007.04.065

**Published:** 2007-09-01

**Authors:** Jenny Crinion, John Ashburner, Alex Leff, Matthew Brett, Cathy Price, Karl Friston

**Affiliations:** a Wellcome Trust Centre for Neuroimaging, UCL, 12 Queen Square, London WC1N 3BG, UK; b MRC Cognition and Brain Sciences Unit, 15 Chaucer Road, Cambridge CB3 2EF, UK

## Abstract

A key component of group analyses of neuroimaging data is precise and valid spatial normalization (i.e., inter-subject image registration). When patients have structural brain lesions, such as a stroke, this process can be confounded by the lack of correspondence between the subject and standardized template images. Current procedures for dealing with this problem include regularizing the estimate of warping parameters used to match lesioned brains to the template, or “cost function masking”; both these solutions have significant drawbacks. We report three experiments that identify the best spatial normalization for structurally damaged brains and establish whether differences among normalizations have a significant effect on inferences about functional activations. Our novel protocols evaluate the effects of different normalization solutions and can be applied easily to any neuroimaging study. This has important implications for users of both structural and functional imaging techniques in the study of patients with structural brain damage.

## Introduction

A necessary step in group analyses of functional magnetic resonance imaging (fMRI) data is precise and valid spatial normalization. The aim of spatial normalization is to establish a one-to-one correspondence between the brains of different individuals, by normalizing each subject to a standard template. This is important for analyzing fMRI data at the between-subject level. Normalization is particularly important when comparisons are made between different groups, especially if one group has structural pathology (e.g., stroke). If brain areas are not properly aligned between individuals, then sensitivity is lost, resulting in false negatives. Conversely, if there are systematic differences in the spatial normalization of the patient group relative to the controls, then true positives may be falsely attributed to differences in functional, as opposed to structural anatomy. The importance of these issues is reflected on the large body of work modeling the effect of lesions on anatomy (Cuadra et al., 2004; Mohamed et al., 2006; Xue et al., 2006; Dawant et al., 2002; Clatz et al., 2005).

Most current spatial normalization methods use automated image-matching algorithms (Ashburner and Friston, 1997; Brett et al., 2002; Toga et al., 2006). However, robust normalization using these methods can be difficult to establish in brains containing focal lesions. These algorithms match brains to a template by minimizing the difference between the subject's image and a template using affine and/or nonlinear warping. The affine approach is a restricted solution that matches the overall size and position of the brain but there is restricted fitting of local structures. Nonlinear warping aligns the sulci and other structures down to a spatial scale specified by the parameterization of the nonlinear warp. Problems can occur when the brain to be normalized has areas of signal loss (e.g., a lesion), which are not in the template. The ensuing mismatch between the brains to be normalized (the source) and the template means that the lesions can confound or bias normalization, usually ‘over-fitting’ the lesioned areas.

An early solution to the over-fitting problem was to constrain the warping of lesioned source images by applying only affine transforms. However, this compromised the fitting of local structures (Warburton et al., 1999a) and led to the development of cost function masking (CFM) (Brett et al., 2001). This removes lesions from the normalization process, thereby allowing nonlinear transformations, while reducing undue effects of the lesion. CFM with nonlinear normalization is superior to affine-only and nonlinear normalization (without CFM) in SPM99 for structural T1 MRI scans; whether it affords improvements in the subsequent analysis of functional imaging is not known. CFM has become a standard procedure for spatial normalization of brain images with focal lesions; however, the method is not without flaws. The brain area under the mask is normalized, but normalization parameters depend largely on homologous non-lesioned regions, making this a poor technique for patients with bilateral pathology. The method also suffers from operator dependence, requiring the user to define the position and extent of the lesion manually. Fully automated brain normalization eschews inter-user variability.

There have been significant advances in the automated normalization schemes in SPM5, which rest on a “unified” model for segmenting and normalizing brains (Ashburner and Friston, 2005). This unified model embodies the different factors that combine to generate an anatomical image, including the tissue class generating a signal, its displacement due to anatomical variations and an intensity modulation due to field inhomogeneities during acquisition of the image. Critically, variations in intensity may be a reasonable model for some lesions, which may render CFM redundant. Thus researchers working with lesioned brains are faced with the choice of using a validated solution: CFM with old normalization schemes (e.g., SPM99), or using more advanced models without knowing if it is necessary to apply CFM. The two unresolved issues that we address in this paper are: first, how do state-of-the-art normalization algorithms compare in terms of their robustness to lesions? And second, what effect do the different spatial normalizations have on the analysis of functional data?

The few studies that have addressed the second question in ‘healthy volunteers’ have been in the context of positron emission tomography (PET) functional imaging studies (Crivello et al., 2002; Kjems et al., 1999; Senda et al., 1998), where little difference has been found among the functional analyses obtained with different spatial normalizations. This may be because of the limited spatial resolution of PET (Crivello et al., 2002). More recently, Ardekani et al. (Ardekani et al., 2004) studied the impact of inter-subject registration on a group analysis of healthy volunteers' fMRI data. They showed that increasing the anatomic accuracy of spatial normalization resulted in significant increases in the sensitivity of activation detection and the reproducibility of activation maps; however, no studies to date have addressed how spatial normalization affects functional data from patients with lesioned brains.

In this paper, we report three experiments that identify the best method for spatial normalization of structurally damaged brains and address the ensuing effects on functional analyses. The objective of the first experiment was to compare quantitatively the anatomic validity of different normalizations (available in SPM5) using normal brains. The objective of the second experiment was to establish whether CFM improves normalization of brains with lesions and whether any improvement depends on the normalization used. To assess this we compared normalizations of lesioned brains using the best normalization from Experiment 1 as a reference. In both experiments we employed anatomical measures of normalization, using landmarks or measures on continuous warps. The objective of the third experiment was to establish and demonstrate a principled protocol, which identifies the best normalization for a particular fMRI paradigm or patient sample.

A key issue is how to assess the quality of a spatial normalization. We have approached this in three ways. In the first experiment, we use anatomical landmarks to compare the success of different normalizations in terms of the spatial dispersion of homologous landmarks following normalization. A normalization with high *face validity* should co-localize landmarks, rendering their spatial variability small. In the second experiment, we compare normalizations of the same brain with and without a simulated lesion and with and without CFM. By doing this we hoped to establish *construct validity*; in the sense that normalization under one construct (lesion or CFM) should correspond to normalization under another. In the third experiment, the *predictive validity* of various normalizations was assessed in terms of whether the structural normalization predicted the outcome of functional analyses. In the next section we describe the general materials and methods used in all three experiments and then detail the specific findings for each experiment in turn at the end.

## Materials and methods

### Data

**Fig. 1:**
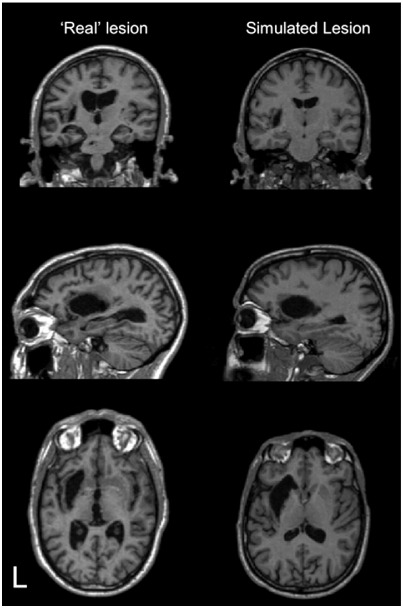
An example of a ‘real’ and a simulated lesioned brain derived from this ‘real’ image used in the anatomical validation: Experiment 2. Image slices shown from top are coronal, sagittal and axial. L = left.

**Fig. 2:**
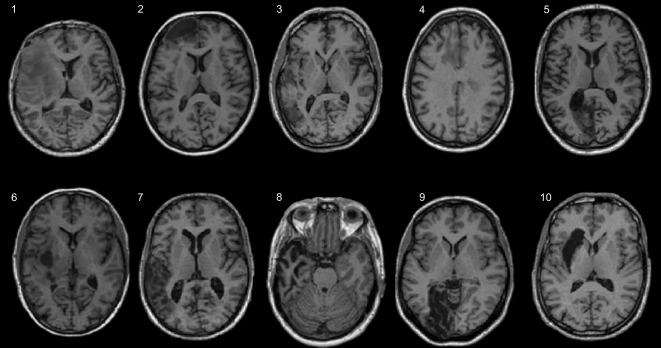
Simulated lesions. Brain images used in the anatomical validation: Experiment 2. Starting from the top left and going clockwise the abnormalities are: 1) left anterior communicating artery stroke, 2) left anterior frontal lesion, 3) left temporo-parietal lesion, 4) multiple areas of cortical damage, 5) left occipitotemporal lesion, 6) left fronto-parietal lesion, 7) left temporo-parietal lesion, 8) left temporal lobe atrophy, 9) left occipitotemporal lesion, 10) left putamen/insula lesion.

In the first two experiments, the data were identical to those reported in Brett et al., 2001. Specifically, these data were T1 MRI images from ten neurologically normal participants before and after a variety of lesions had been introduced to simulate a wide range of structural pathologies, including artefacts with high and low signal intensity (simulated lesion images). These simulated lesion images are synthetic images created by Brett et al. (2001). Full details of how the simulated lesions were created can be found in Brett et al. (2001) page 492. Essentially the method involved first creating a binary lesion definition image (ROI of lesioned tissue) using T1-weighted MRI images of ‘real’ patients’ brains with a variety of lesions. These lesion images were then inserted into co-registered T1-weighted MRI images from subjects with no neurological abnormality (normal brains). An example of a ‘real’ lesioned brain and its simulated lesion image is shown in Fig. 1. Additional examples of the simulated lesion images used in Experiment 2 are shown in Fig. 2. In the third experiment, we used the fMRI data from a study of speech comprehension in 18 chronic stroke patients (Crinion and Price, 2005).

### Normalization procedures

For all normalizations we used the options in SPM5 (http://fil.ion.ulc.ac.uk/spm); each scan was normalized to the T1 MNI templates supplied with SPM5. The standard SPM5 normalization scheme minimizes the sum of squared difference between the image to be normalized, and a linear combination of one or more template images. The first step of the normalization is to determine the optimum twelve-parameter affine transformation. Initially, the registration is performed by matching the whole of the head (including the scalp) to the template. The registration then proceeds by matching the brains using an appropriate weighting on template voxels. A Bayesian framework is used, such that the registration searches for a solution that maximizes the posterior probability of the warping parameters (Ashburner et al., 1997).

The affine registration is followed by estimating nonlinear deformations, in terms of a linear combination of three dimensional discrete cosine transform (DCT) basis functions (Ashburner and Friston, 1999). The default options encode deformation fields with 1176 parameters (the coefficients of the deformations in three orthogonal directions). The matching involves simultaneously minimizing the bending energies of the deformation fields (prior term) and the residual squared difference between the images and template (likelihood term) to provide the conditional (posterior) estimate of the deformation.

The newer alternative normalization in SPM5 combines segmentation, bias correction and spatial normalization in the inversion of a single unified model. Estimating the model parameters (to give a maximum *a posteriori* solution) involves alternating among classification, bias correction and registration steps. This approach affords better results than serial applications of each component because conditional dependencies among the model parameters are modeled properly; i.e., registration and bias correction help the tissue classification, and the tissue classification helps the registration and bias correction (Ashburner and Friston, 2005). The multiple Gaussians that are used to model the intensity distributions of the different tissue classes help model the lesions. The assumption of non-Gaussian intensity distributions may allow healthy white matter to be modeled by one Gaussian intensity distribution, and lesioned white matter to be modeled by another. The inclusion of bias correction in the unified generative model is also important in the present context because it may model lesions and therefore suppress their effects. This is because the model includes an inhomogeneity field, which models variations in the intensity of the image over space. To the extent that lesions can be modeled as large-scale intensity variations, unified normalization may implicitly model lesions (i.e., perform an automatic cost function masking). Further details of the unified segmentation model can be found in Appendix A.

For both standard and unified normalization the priors on distortions were examined at three levels (low, medium and high). These priors enforce smooth warps and regularize the normalization to prevent over-fitting (see also Salmond et al., 2002). The amount of regularization (lambda) varied by a factor of ten so that ten times as much regularization was used for high regularization as was used for medium regularization, and ten times as much regularization was used for medium regularization as it was for low regularization. This regularization factor can be regarded as increasing the precision (i.e., inverse variance) of the bending energy priors on the deformation, relative to the likelihood (sum of squared difference between the normalized and observed images) under Gaussian assumptions about noise. For a formal description see Eq. 5.11 in Ashburner and Friston, 2007. This gave us six different normalizations; three different regularizations of conventional and unified inversions (for completeness we also examined affine-only transforms; the results we obtained for affine-only were very similar to those for high regularization, which is consistent, because in the limit of very high regularization nonlinear deformations become affine). All other normalization parameters were held constant (e.g., 16 nonlinear iterations).

The effect of CFM was also examined for the six different normalizations. This gave us in total 12 different normalizations; three different regularizations of conventional and unified inversions with and without CFM. Spatial normalization algorithms use the differences in intensity values of the source image and template to derive a mathematical measure of mismatch between the images—a “cost function.” CFM excludes the lesioned area from the calculation of image difference thereby restricting the cost function to areas of brain outside the abnormality. In this study, we have implemented cost function masking using a simple binary mask image, which has values of one outside the lesion, corresponding to normal brain and zero within the lesion. The user first defines the area of the lesion on the structural image. The lesion definition is inverted and expanded to account for the effect of smoothing during normalization. When the mask is applied to the source image the lesion no longer influences the optimization of the spatial normalization parameters. Therefore there is no attempt to minimize image differences in the area of the lesion, and the lesion does not bias transformations elsewhere in the brain. Note that masking the abnormal region does not mean that areas under the mask remain untransformed; rather, a continuation of the solution for the unmasked portions of the image is applied to the areas under the mask. This continuation will be constrained to be smooth by the use of the nonlinear regularization term in the normalization process.

Methods for normalization with cost function masking are implemented in the current software. For example, there are tools for the creation of lesion definition images in MRIcro (http://www.psychology.nottingham.ac.uk/staff/cr1/mricro.html). The standard distribution of SPM5 supports the use of source masking images in normalization, when “object masking” is enabled in the Spatial Normalization section of the program defaults.

### Statistical models

#### Experiment 1

**Fig. 3:**
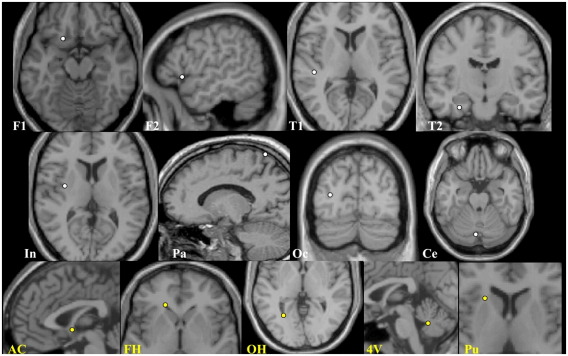
Experiment 1: anatomical landmarks. Location of anatomical landmarks shown on an exemplar brain: cortical landmarks are shown in white (*n* = 16, 8 each hemisphere): 2 frontal = F1, F2; 2 temporal = T1, T2; insula = In; parietal = Pa; occipital = Oc; cerebellum = Ce. Subcortical landmarks are in yellow (*n* = 8, 2 midpoint, 3 bilateral): Anterior commissure = AC; frontal horn = FH; occipital horn = OH; 4th ventricle = 4V; putamen = Pu.

In Experiment 1, the ten original images (no lesions) were labeled with 24 anatomical landmarks by a neurologist (APL, see Fig. 3). The success of the normalization was based on the dispersion (root mean square; RMS displacement) over subjects of each landmark in anatomical space following normalization. The 3D variance–covariance matrix was computed for each normalization using the deformation fields. The resulting variances were used to compute the RMS dispersion and compared using SPSS 12, using a univariate analysis for a fully balanced 2 × 3 factorial design as follows: Factor 1: normalization (unified vs. standard). Factor 2: regularization (low vs. medium vs. high), as outlined above. The statistical criterion was *p* < 0.05.

#### Experiment 2

In Experiment 2, the robustness of the normalizations to the introduction of lesions was assessed by comparing normalizing deformations (i.e., warps) for brains with simulated lesions to those obtained for the same brains without lesions. To assess robustness we used a RMS displacement measure over all voxels to summarize the normalization of lesioned brains, relative to the true normalization. Clearly, we do not know the true normalization, so we used the best normalization of the previous experiment, applied to a non-lesion version of each brain. The best normalization was unified normalization, with default [medium] regularization. The measure of the difference between these two deformations was the root mean squared displacement (RMSD) of one deformation field relative to another:

$$\text{RMSD}=\sqrt{\frac{1}{N}\sum_{i=1}^{N}d_{i}^{2}}$$

where *d*_*i*_ is the distance between the same voxel *i* in both deformations and *N* is the number of voxels (the whole brain). In summary, the RMSD encodes the precision of the normalization, relative to the best normalization of the un-lesioned brain; a small RMSD means a good normalization which is not unduly sensitive to the lesion. These measures were obtained with and without CFM to see if CFM made the normalizations more robust to lesions.

We computed an RMSD measure for each image (*n* = 10) for each normalization with and without CFM. We treated the RMSD as a dependent variable in a three-way ANOVA in SPSS 12, using a univariate model with a fully balanced 2 × 3 × 2 factorial design: Factor 1: normalization (unified *vs.* standard); Factor 2: regularization (low vs. medium vs. high). Factor 3: CFM (with vs. without).

#### Experiment 3

In Experiment 3, the effect of different normalizations on functional anatomy was characterized using real patient fMRI data. Eighteen patients (12 males, mean 62 years, s.e. 2.7) with left hemisphere stroke (mean 45 month, s.e. 17.9 months, post infarct) participated in an fMRI study of auditory speech comprehension. Nine patients had a left hemisphere lesion sparing the temporal lobes, the remaining nine patients had temporal lobe damage. They all had English as their first language and were right-handed.

They listened to two sorts of auditory stimuli while in the scanner: stories and time-reversed versions of the same stimuli (baseline condition). The order of presentation was randomized, both within and between subjects. Presentation was binaural, with the volume set at a comfortable level for each subject. The subjects were asked to simply listen and try to understand the stories. The patients were aware that the baseline was unintelligible but were asked to pay attention to the sounds. The details of this study can be found in Crinion and Price (2005).

In this experiment we analyzed the same data in SPM5, but applied different normalizations to the contrast images produced by a conventional first (within-subject) level analysis. This was achieved by co-registering each patient's structural (T1 MRI) scan to their mean realigned fMRI data. Both co-registration and realignment of the fMRI data use affine (3D rigid-body) transformations, under the assumption that the shape of each subject's brain does not change substantially. The structural scan was then normalized using the procedures in Experiment 2. Each subject's contrast (stories vs. baseline) was then normalized using the different deformation fields to produce twelve (2 × 3 × 2) contrast images for each of the 18 patients. We then assessed the effects of normalization in SPM using a series of two-sample *t*-tests: Test 1: main effects of normalization (unified vs. standard); Test 2: main effect of CFM (with vs. without) and Test 3: main effect of regularization (low vs. medium and medium vs. high). Notice that these effects represent the interaction between normalization and functional responses because the contrast encoded the activation elicited by listening to stories; in other words, these analyses test for the effect of normalization on regionally specific activations.

All analyses were thresholded at *p* = 0.001 (uncorrected) and masked exclusively for the main effect of the contrast (stories vs. baseline) revealed by a one-sample *t*-test. This meant that we confined our inference about normalization effects to the bilateral temporal and frontal cortices that showed functional effects.

## Results

### Experiment 1: comparing different normalizations

We can assume that the errors our neurologist, ‘anatomical expert’, made were similar across the ten subjects (although the error introduced will not be the same for each landmark as some were harder to define than others). The level of the error should not affect the relative or ordinal performance of the normalizations (it will only affect the absolute RMS values). To quantify intra-rater error we used the standard deviation of the difference between left and right landmarks. A perfect rater (assuming absolute symmetry of the brain) should score zero. Our rater had a standard deviation of 1.4 mm over all twelve landmarks.

**Fig. 4:**
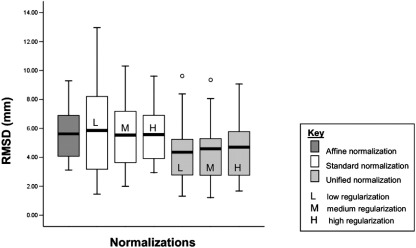
Experiment 1: comparing different normalization algorithms. Plot of the root mean square (RMS) values in millimeters comparing the normalizations of the group of ten normal images for all the cortical (*n* = 16) and subcortical (*n* = 6) landmarks. From the left of the image the first (dark gray) box-plot shows RMS values for the affine-only solution *(mean—5.71, s.d—1.76)*, then next the three (white) standard solutions with low (L) *(mean—6.14, s.d—3.*2), medium (M) *(mean—5.74, s.d—2.48),* and high (H) *(mean—5.67, s.d—1.88)* regularizations. The subsequent 3 box-plots in light gray show RMS values for unified solutions with low *(mean—4.50, s.d—2.17),* medium *(mean—4.49, s.d—2.1)* and high *(mean—4.67, s.d—2.04)* regularizations. The black line in each box-plot indicates the group mean for each normalization solution. The open circle indicates a group outlier.

For the ten normal brains, there was a main effect of normalization with the unified model giving significantly better co-localization, as measured by smaller RMS dispersion over all 24 landmarks (*p* = 0.001). Specifically, the mean RMS displacements were 4.55 mm; (s.d. = 2.07) for unified normalizations and 5.82 mm, (s.d. = 2.37) for standard normalizations. There was no main effect of regularization and no significant interaction between regularization and normalization. The mean spatial dispersion and standard deviations for each normalization scheme are illustrated in Fig. 4. The best normalization was unified normalization, with default [medium] regularization.

These results suggest that the unified model gives the most precise registration of normal brains. Altering the regularization has little overall effect on the precision of normalization, irrespective of the procedure used. Interestingly, the results illustrate that in SPM5 the affine registration seems to be more accurate than the standard nonlinear normalization with medium and low regularization. When high regularization is used the result is a transform that is closer to affine and the results for high regularization and affine-only transforms converge. For the unified model, the parameterization of the deformations is similar but registration is limited to the brain only. Therefore, the effect of regularization is different, with variance increasing with increasing levels of regularization again converging on the affine-only performance levels.

The surprising observation that affine is better than standard nonlinear normalizations may be the result of (i) using a low-dimensional deformation model and/or (ii) including the skull in the normalization procedures. The evaluations of SPM spatial normalization by Hellier et al. (2003), in terms of RMS errors, used skull-stripped images and showed nonlinear registration performed pretty well. We did not use skull-stripping, which means that the algorithm may have tried hard to register the signal in the scalp, at the expense of reducing the accuracy of the fit in the brain.

While the unified model gives a superior solution for normal brains, it may fail to prevent anatomically unlikely transformations in lesioned brains (as the model has no specific priors for lesioned tissue). This possibility was investigated in Experiment 2, in which we looked for an effect of normalization, regularization and CFM on the RMSD measure of normalization performance with and without lesions.

### Experiment 2: the effects of lesions on normalization

In lesioned brains there was a main effect of normalization with the unified model giving significantly better results (smaller mean RMSD) overall (*p* = 0.0001); unified segmentation without CFM; mean = 1.64 mm, s.d. = 0.74; standard solutions without CFM; mean = 5.29 mm, s.d. = 2.14. There was also a main effect of regularization (*p* = 0.0001) with medium and high levels giving significantly better results than low regularization (*p* = 0.05). The interaction between regularization and unified vs. standard was significant (*p* = 0.001), with high and medium regularization affording superior solutions under unified normalization. There was no main effect of CFM and no significant CFM interactions with regularization or normalization model.

**Fig. 5:**
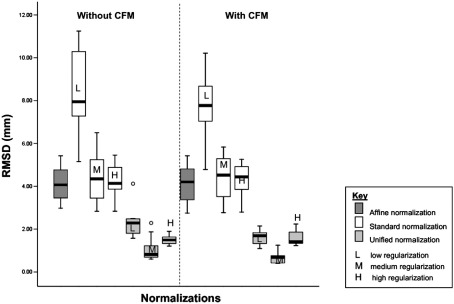
Experiment 2: Normalization of lesioned brains with and without CFM. Plot of the root mean squared difference (RMSD) values in millimeters for the 14 normalizations of the group of ten simulated lesion–normal brain pairs. From the left of the image the first (dark gray) box-plot shows RMS values for the affine-only solution *(mean—4.1, s.d—0.86),* then next the three (white) standard solutions with low (L) *(mean—8.36, s.d—1.82),* medium (M) *(mean—4.4, s.d—1.12)* and high (H) *(mean—4.31, s.d—0.77)* regularizations. The subsequent 3 box-plots in light gray show RMS values for unified solutions with low (L) *(mean—2.33, s.d—0.71),* medium (M) *(mean—1.09, s.d—0.58)* and high (H) *(mean—1.52, s.d—0.25)* regularizations. The 7 subsequent box-plots, after the dotted line with shaded background, show the same normalization solutions with cost function masking (CFM): affine *(mean—4.13, s.d—0.88),* standard solutions with low (L) *(mean—7.82, s.d—1.54)*, medium (M) *(mean—4.35, s.d—1.07)* and high (H) *(mean—4.35, s.d—0.77)* regularizations, unified solutions with low (L) *(mean—1.62, s.d—0.32)*, medium (M) *(mean—0.72, s.d—0.31)* and high (H) *(mean—1.57, s.d—0.34)* regularizations. The black line in each box-plot indicates the group mean for each normalization solution. The open circle indicates a group outlier.

Fig. 5 shows the results of the root mean squared difference (RMSD) values for the normalizations of the group of ten simulated lesion–normal brain pairs. The first four plots show the RMSD values for the affine-only and then standard nonlinear normalizations, without CFM. The values are relatively high, compared to the unified values shown in the next three plots. There is also a considerable spread of the values across images, reflecting a high variability of normalization performance across images and lesions. The figure therefore shows variability in normalization performance across images. The simulated lesions used in Experiment 2 covered a wide set of brain regions. Therefore our whole brain analysis was influenced by many different local areas of lesioned brain. We did not assess the interaction between the lesion location and the success of the normalization algorithm because this would entail the comparison of normalization in groups of patients with similar lesions within group. These data are not easy to acquire because no two lesions are ever the same. The last seven plots are from the same normalizations but with CFM. These are remarkably similar to those without CFM. The ideal normalization method would provide low RMSD values, with a narrow spread of values across images. On these criteria, the unified models perform well, with high regularization providing the tightest distribution of values across images.

**Fig. 6:**
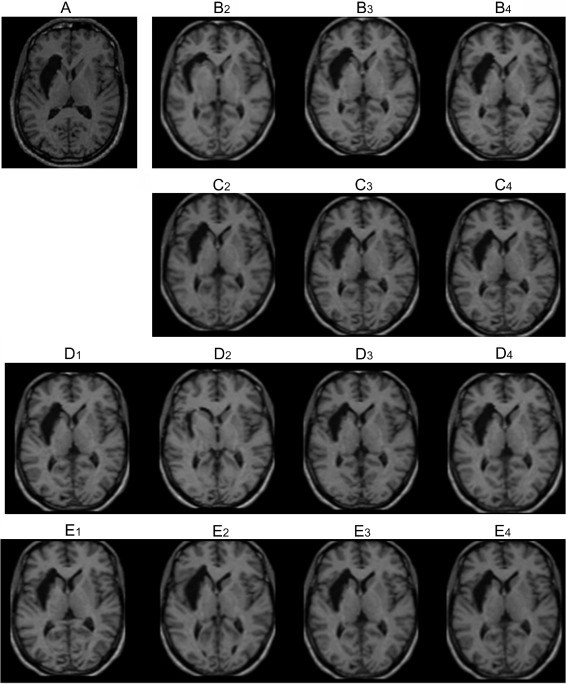
The 14 normalization solutions for an individual brain, simulated brain lesion number ten from Fig. 2. The images show the brain after affine-only normalizations, standard nonlinear normalizations and unified models, with and without cost function masking. (A) The un-normalized T1 MR image of simulated lesion 10. (B) The unified models with low, medium and high regularizations. (C) The same unified models as in B above with CFM. (D) The affine-only (1) and standard normalizations with low, medium and high regularizations. (E) The same normalizations as in row D above with CFM.

The results of the different normalizations of an individual lesioned brain are illustrated in Fig. 6. Image A shows the un-normalized image of the simulated lesion, derived from a patient with a left hemisphere stroke. The numbers indicate the level of regularization used i.e., 2—low, 3—medium, 4—high. The letters indicate the algorithm used i.e., B and C = unified models without and with CFM respectively, D and E = standard solutions without and with CFM. It is clear that low regularization without CFM, images B2 and D2, provided a poor solution, shrinking the lesion and inducing marked distortions nearby. With these solutions CFM did visibly improve the fit, reducing the influence of the lesion on the nonlinear transforms and less ‘crushing’ of the lesion, as shown in images C2 and E2. The affine-only normalizations, shown in C1 and D1, appear to be robust to the effects of the lesion but are still influenced by it. This can be difficult to see by eye but from the analyses we know, on average, the affine-only solution provides a less accurate match of local brain detail in the order of 2 to 3 mm than the unified segmentation solutions as shown in images B and C. As illustrated in Fig. 6, qualitative judgments by eye of ‘goodness’ of normalization, even of lesioned brains, are not a sensitive or reliable approach for choosing the most accurate and valid solution.

These results suggest that unified models give the most spatially precise registration of lesioned structural images. Regularization also has a significant effect on the normalization of lesioned images. In lesioned brains, increasing the regularization levels (by an order of magnitude) gave a better solution. CFM offers no advantage when using these regularization levels.

### Experiment 3: the impact of normalization on fMRI results

**Fig. 7:**
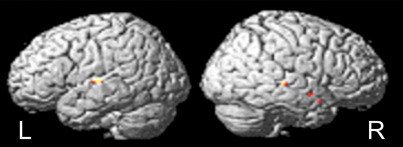
Experiment 3: fMRI results. Regional activation for unified > standard normalization solutions. For the group of 18 stroke patients, results are shown in color rendered onto the SPM5 single-subject brain template. In the left (L) hemisphere there was one peak in the middle superior temporal gyrus (coordinate *x* = − 58 *y* = − 14 *z* = 6; *z* score = 3.98). In the right (R) hemisphere there were 3 main peaks in the posterior, (*x* = 50 *y* = − 28 *z* = 0; *z* score = 3.63), middle (*x* = 58 *y* = − 2 *z* = − 12; *z* score = 3.72) and anterior (*x* = 44 *y* = 8 *z* = − 16; *z* score = 3.84) superior temporal sulcus.

The activation associated with speech comprehension in 18 patients was significantly affected by the normalization used. We confined our inference about normalization effects to the cortices that showed functional effects for the group. There were no significant effects at *p* = 0.05 corrected outside the mask for the main effect of the contrast (stories vs. baseline) as tested by a one-sample *t*-test. There were no significant effects of regularization or CFM on the fMRI results from this group of stroke patients; improved inference about functional anatomy was afforded primarily by the use of a unified model, which implicitly, may model lesions more effectively. The unified solution increased fMRI activations bilaterally in the superior temporal lobes. In the left hemisphere there was one peak in the middle superior temporal gyrus (coordinate *x* = − 58 *y* = − 14 *z* = 6; *z* score = 3.98); while on the right there were three main peaks in the posterior, middle and anterior superior temporal sulcus (*x* = 50 *y* = − 28 *z* = 0; *z* score = 3.63; *x* = 58 *y* = − 2 *z* = − 12; *z* score = 3.72; *x* = 44 *y* = 8 *z* = − 16; *z* score = 3.84). See Fig. 7.

The results from this experiment illustrate that different normalizations can have a significant effect on fMRI activations from patients with brain lesions. In our fMRI study of speech comprehension in stroke patients, unified segmentation offered the best solution, co-localizing activations in the bilateral superior temporal lobes. Note that the only way that normalization can affect the regional activations is to move them around so that they co-localize and produce a bigger statistic at the between-subject level. This means that an improvement in the *t*-statistics above (i.e., main effect of normalization) can only be due to better co-registration of functional anatomy and, implicitly, robustness to any affect of lesions on that co-registration.

## Discussion

The problem addressed in this paper is the tendency for automated warping algorithms to produce inappropriate solutions when normalizing brains with lesions. These problems occur when matching brains to a template that does not have a lesion. Most previous studies that have used automated normalization for brains with lesions have used affine-only transformations (Price et al., 1998; Mummery et al., 1998; Warburton et al., 1999a,b) or nonlinear deformations with CFM (Leff et al., 2002; Sharp et al., 2004; Crinion et al., 2006). Here, we compared the effects of using different models for spatial normalization, with and without CFM. We found that unified models produced significantly better results, both in terms of anatomical co-localization, and the effect this has on detecting functional activation. Our results illustrate for the first time that the normalization model can have a significant effect on both the anatomical precision of normal control data, lesioned patient data (structural MR data) and size of fMRI (functional magnetic resonance imaging) effects in a group of chronic stroke patients.

The objective of inter-subject registration is to bring homologous structures into alignment. Its accuracy can be measured either in terms of minimizing the difference of each brain relative to a gold standard template or in terms of minimizing the dispersion between the brains that are being aligned. In this paper, we chose to measure registration accuracy in terms of minimal dispersion because landmark identification was easier on the raw images (than on the template brain that is averaged over large numbers of subjects). We then selected the most accurate registration as the gold standard in the subsequent analyses. This is an operational definition of gold standard and we do not mean to imply that there is no better solution. However, the protocol we have provided is novel and can be used to evaluate the effects of different normalizations on patient fMRI studies. We have only tested solutions within SPM5 but the protocol can be applied to any neuroimaging software platform. The protocol is purely operational in nature and rests on model selection using the null hypothesis that different normalizations have no effect on the expression of regional effects at the group level. This provides researchers with a principled way of choosing between different normalization algorithms and selecting the best, should there be any difference, for their own functional imaging dataset.

Contrary to popular belief, our analyses suggest that the choice of normalization solution can affect ones fMRI results. This is particularly important when comparisons are made between different groups, especially if one group has structural pathology (e.g., stroke). If brain areas are not properly aligned between individuals, then sensitivity is lost, resulting in false negatives. In our fMRI study of speech comprehension in stroke patients, unified segmentation offered the best normalization solution, co-localizing activations in the bilateral superior temporal lobes and furnishing increased sensitivity to activation in this region, in the patients. This bilateral activation of the superior temporal lobe to meaningful speech may have been missed in previous studies that used suboptimal normalization schemes. Conversely, if there are systematic differences in the spatial normalization of the patient group relative to the controls, then differences in fMRI effects, on comparing the two groups, may be falsely attributed to differences in functional, as opposed to structural anatomy. This may go someway towards explaining the confusion in the literature, where some differences in the findings between research groups may reflect differences in normalization algorithms rather than differences in functional anatomy.

Our results suggest that normalization using the unified models is superior to standard nonlinear approaches (with CFM) for two main reasons. First, unified models improve the quality of the normalization, both for normal brains and for lesioned brains, without causing aberrant distortions in the lesioned image. Inversion of the unified models proceeds by iterating the following steps: (i) registration, without the bias from fitting the skull and scalp; (ii) classification of gray, white matter and cerebrospinal fluid according to tissue probability maps; and (iii) non-Gaussian intensity correction. The latter component means that the solution is less susceptible to error or bias from a lesion than standard nonlinear and affine-only methods. Second, unified segmentation is fully automated making it less time consuming and more objective than CFM.

Even for unified models, normalizations performed on lesioned brains are likely to be less successful than those on normal brains. Lesions can cause intensity changes to existing tissue (e.g., stroke), or displace existing tissue (e.g., tumor). Therefore normalization error will be higher for damaged than undamaged brains. For a lesioned brain, a proportion of brain (the lesioned signal) cannot be accurately matched, as shown by the non-zero RMSD values for the brains with simulated lesions. This error will be maximal within the lesion but can also affect the normalization of peri-lesional brain. Despite this, the error is surprisingly small, in the order of a 1.6 mm difference between normal and lesioned brains (Fig. 5). This is much smaller than the typical smoothing kernel used in fMRI group studies (8 mm^3^). For these reasons, when using the unified method it should not be considered problematic to compare activations from a group of normal subjects with those from lesioned brains. However, researchers carrying out studies, where the regional expectation for task-related activation is in peri-lesional tissue, might consider analyzing non-normalized data in the first instance (i.e., a series of single-subject analyses) and ensure that the group results are internally consistent with the series of case studies.

Future developments using the unified models framework will allow an extension to include lesion priors, whether based on the approximate spatial location of the lesion or by using multi-spectral data (e.g., from a registered T1 and T2* image) to increase further the anatomical precision.

## Conclusion

In this paper, we have presented and evaluated new automated techniques available in SPM5 for spatial normalization of structural and functional brain images in both normal subjects and patients with brain lesions. The results suggest that the unified models for anatomical variation provide better and more reliable matching to a standard template than the commonly used alternatives. We propose that the unified solution should be used routinely, when normalizing brains containing areas of abnormal signal. The novel protocols we provide can be applied to any functional or structural brain imaging dataset allowing researchers and clinicians working in neuroimaging to easily evaluate for the first time the effects of different normalization solutions on their own data.

## Software note

Methods for normalization with the unified model are implemented in current SPM5 software. There is a step by step tutorial on creating and using segmentation images in spatial normalization with SPM5 available freely at http://www.fil.ion.ucl.ac.uk/spm/doc/manual.pdf.

## Appendix A Unified models

The unified model combines tissue classification, bias correction and nonlinear warping into the same probabilistic generative model. Image intensities are modeled by mixtures of Gaussians (MOGs). Within a simple MOG, the prior probability of an observation being drawn from a particular Gaussian is given by a mixing proportion. In the unified model, these priors are encoded by deformable tissue probability maps, generated from the averages of affine registered and tissue classified images of 451 subjects. These maps represent the probabilities of finding gray matter, white matter, CSF and “other”' tissues at each voxel. The tissue probability maps are deformed by an affine and nonlinear displacements in the three orthogonal directions, parameterized by about a thousand low-frequency cosine transform basis functions. The resulting displacements are regularized by minimizing the bending energy of the displacement fields. Generation of the spatially normalized images requires the inverses of these deformations. This is achieved by a procedure that re-parameterizes the deformations by very fine piecewise affine transforms (arranged as tightly packed tetrahedra). Inverting a deformation involves finding which voxels of the tissue probability map template fall inside each projected tetrahedron and assigning them mappings according to the affine transform of that tetrahedron.

In this paper, the unified model used the default number of Gaussians; mixture of two Gaussians is used to model each of the intensity distributions of gray matter, white matter and CSF, whereas a mixture of four Gaussians models the intensity distribution of the “other” tissues. No exploration of the effect of varying the number of Gaussians was performed, and nor was any model selection for determining the optimal number. Although not verified in any way, one can conjecture that this number of Gaussian mixtures can model lesions, specifically in the “other” class, which does not contribute to the normalization parameter estimates. More flexible models for the intensity distributions of various tissues are obtained by using more Gaussians, which make it easier to account for intensity variability resulting from pathology. Intensity non-uniformity is modeled by extending the MOG to include a smooth function over the voxels in the image, which scales the mean and standard deviation of each Gaussian of the MOG.
